# Pathological social withdrawal in autism spectrum disorder: A case control study of hikikomori in Japan

**DOI:** 10.3389/fpsyt.2023.1114224

**Published:** 2023-03-23

**Authors:** Mari Yamada, Takahiro A. Kato, Ryoko Inoue Katsuki, Hideki Yokoi, Miki Igarashi, Yoko Komine, Yukinori Kamata, Nobumasa Kato, Akira Iwanami, Haruhisa Ohta

**Affiliations:** ^1^Department of Psychiatry, Showa University of Medicine, Tokyo, Japan; ^2^Department of Neuropsychiatry, Graduate School of Medical Sciences, Kyushu University, Fukuoka, Japan; ^3^Department of Pediatrics, Graduate School of Medical Sciences, Kyushu University, Fukuoka, Japan; ^4^Medical Institute of Developmental Disabilities Research, Showa University, Tokyo, Japan; ^5^Department of Clinical Psychology, University of the Sacred Heart, Tokyo, Japan; ^6^Research Wing, Neuropsychiatric Research Institute, Tokyo, Japan

**Keywords:** autism spectrum disorder (ASD), hikikomori (social withdrawal), uric acid (UA), social isolation, modern type depression, social anxiety, sensory sensitivity

## Abstract

**Introduction:**

Hikikomori, a form of pathological social withdrawal, has been suggested to have comorbidity with autism spectrum disorder (ASD). This study aimed to clarify how characteristics of ASD are associated with hikikomori.

**Methods:**

Thirty-nine adult male patients with a diagnosis of ASD attending our outpatient clinic for neurodevelopmental disabilities were subjected to a structured interview regarding social withdrawal, various self-administered questionnaires, and blood tests. Through structured interviews, the subjects were divided into two groups: (Group 1) ASD with hikikomori condition and (Group 2) ASD without hikikomori condition. Sixteen subjects qualified as hikikomori and 23 subjects qualified as subjects without hikikomori. Age, sex, autism spectrum quotient (AQ), Autism Diagnostic Observation Schedule (ADOS), and FIQ were matched.

**Results:**

Compared to non-hikikomori controls, hikikomori cases were likely to have stronger sensory symptoms, lower uric acid (UA) (*p* = 0.038), and higher rates of atopic dermatitis (*p* = 0.01). Cases showed more severe depressive and social anxiety symptoms based on self-rated scales: Patient Heath Questionnaire 9 (PHQ-9) (*p* < 0.001) and Liebowitz Social Anxiety Scale Japanese Version (LSAS-J) (*p* = 0.04). Tarumi's Modern-Type Depression Trait Scale (TACS-22), which measure traits of Modern-Type Depression (MTD), were significantly higher in cases (*p* = 0.003).

**Conclusion:**

The present study has suggested that ASD patients with hikikomori were more likely to have higher sensory abnormalities, comorbid atopic dermatitis, lower UA, stronger depressive, and anxiety tendency. Evaluating and approaching these aspects are important for appropriate interventions in ASD with hikikomori. Further investigations should be conducted to validate our pilot findings.

## 1. Introduction

The “hikikomori” syndrome is a form of prolonged and severe social withdrawal or social isolation that continues for more than 6 months (1), first defined and studied in Japan approximately two decades ago. The population of the syndrome is estimated to be more than a million people in Japan, and although the number is not yet aware, many people with hikikomori exist throughout the world (2–4), making the syndrome a growing issue.

In prior research examining hikikomori, various psychiatric disorders have been suggested to comorbid hikikomori (1, 5), including anxiety disorder, depression, personality disorder, and autism spectrum disorder (ASD).

Autism spectrum disorder is characterized by the presence of persistent difficulties in social communication, restricted and repetitive patterns of behavior, interests, or activities, and hypersensitivity to noise, touch, or light. Hypothetically, these characteristics may fundamentally lead to hikikomori easily. Kondo et al. reported that 31.8% of 183 hikikomori sufferers who attended mental health welfare centers had neurodevelopmental disorders (5). Tateno et al. reported that psychiatrists and pediatricians regarded one-fifth of their hikikomori patients as F8 (disorders of psychological development) such as pervasive developmental disorder (PDD) using the International Statistical Classification of Diseases and Related Health Problems (ICD)-10-based diagnosis (6).

In 2020, Katsuki et al. has shown the possible relationship between autistic tendency and hikikomori (7). One hundred and three hikikomori cased recruited through their mood disorder/Hikikomori Clinic at Kyushu University, compared to 221 clinical controls without hikikomori conditions in series of self-rated scales, including the Japanese version of the Autism-Spectrum Quotient (AQ-J) (8). Interestingly, hikikomori cases were more likely to have higher autistic tendency based on the AQ-J. In addition, hikikomori sufferers with high autistic tendency had significantly lower self-esteem and higher complaint tendencies, and such two aspects could be regarded as traits of Modern-Type Depression (MTD) (9). Thus, previous studies suggested that ASD and hikikomori are closely related and that ASD with hikikomori may have unique characteristics. However, little is known about the feature of clinically diagnosed ASD with hikikomori syndrome, compared to non-hikikomori ASDs.

Hikikomori may be caused not only by psychological and social factors, but also by biological factors. A large nationally population-based study has suggested that social isolation increase the risk of inflammation (C-reactive protein, CRP), and the effect of social isolation on hypertension exceeded that of clinical risk factors such as diabetes in old age (10). Hayakawa et al. recently reported that individuals with hikikomori show lower serum uric acid (UA) levels in men and lower high-density lipoprotein cholesterol (HDL-C) levels in women compared with healthy volunteers. Furthermore, avoidant personality traits were negatively associated with UA in men, and positively associated with fibrin degeneration products (FDP) and high-sensitivity C-reactive protein (hsCRP) in women among non-hikikomori volunteers. This study suggested the existence of blood biomarkers for hikikomori. There are great interests of whether these biomarkers are reproducible in ASD with hikikomori.

Another growing interest was common comorbidities of ASD. Atopic dermatitis has often been reported as a common comorbidity of psychosocial disorders such as depression, anxiety (11), and even ASD (12). Kato et al. reported high comorbidity of atopic dermatitis with hikikomori (1). In this study, we hypothesized a possible connection of atopic dermatitis, hikikomori, and ASD.

Among the various psychiatric disorders that cause hikikomori, there has yet been only a few studies that have examined the relationship between hikikomori and each disorder. Therefore, in the present study, we sought to examine the relationship between ASD and hikikomori, biologically and psychosocially. Additionally, in this study ASD has been strictly diagnosed, based on the strict diagnostic system using Autism Diagnostic Observation Schedule (ADOS), Wechsler Adult Intelligence Scale (WAIS), and several other tests at Showa University Medical Institute of Developmental Disabilities Research Center.

## 2. Methods

### 2.1. Setting and procedures

Recruitment was conducted through the adult developmental disorders outpatient basis of Showa University Karasuyama Hospital. All participants signed a written consent prior to completing the questionnaires and testing in the study, which was approved by the ethics committee of the Faculty of Medicine of Showa University. In concern of the sex difference of the feature of ASD (13), participants were limited to males. Age limitation was set to above 18 years old. Data were collected between April 2019 and March 2020.

In terms of ASD diagnosis, all patients were asked to complete an interview sheet during their initial visit, prior to their clinical examination. The interview sheet addressed the following five areas: 1. Major complaint, 2. History of visits to medical and educational organizations/consultation services, 3. Problems *in utero* or during infancy, 4. Developmental delays (walking and language), and 5. Education and occupation of the patient and their parents. The patients were also required to bring their school records from elementary school through high school, and their maternal and child health handbook. The maternal and child health handbook, which is provided by the local government office in Japan, includes records of pregnancy, childbirth, and the neonatal and infant periods. A further diagnostic assessment of all patients was performed by a team of experienced psychiatrists. The assessment comprised two detailed interviews of the patients about the development and behavior from infancy to adolescence (1. developmental history, 2. Present illness, and 3. medical history), and family history. The patients were also asked to bring suitable informants who knew the patient during their early childhood. At the end of the clinical interview, the patients were diagnosed according to the DSM-5 diagnostic criteria for ASD by a psychiatrist, after the psychiatrists on the team reached a consensus. Although diagnosis itself of psychiatric disorders is error-prone, we consider diagnostic error to be relatively low.

We recruited 39 adults with a primary diagnosis of ASD. All subjects underwent AQ-J. Among the 39 individuals with ASD, we administered the ADOS (14, 15) to 26 individuals, all of whom met the diagnosis for ASD. WAIS-III was performed for all but one of the 39 patients.

Patients with a primary diagnosis of schizophrenia, socialphobia, major depressive disorder, and obvious intellectual disability were discluded, considering the influence toward social withdrawal. Two patients had an FIQ of 75 or less (FIQ = 68, 69), while most had no intellectual disability. As of previous psychiatric comorbidity, two patients had a controlled state of bipolar disorder -II, two had attention deficit hyperactivity disorder (ADHD), and one had a controlled state of epilepsy. These patients were included in this study because their comorbidities were controlled and their primary medical conditions were mainly due to ASD symptoms.

Non-psychiatric comorbidities were surveyed. The presence of absence of atopic dermatitis was specifically checked and statized.

Cases consisted of ASDs with current social withdrawal and non-social withdrawal. We conducted the semi-structured interview for hikikomori made by Kato et al., to evaluate period and severity of social withdrawal (16). Interviews were conducted by psychiatrists with extensive clinical experience. Social withdrawal was defined as spending most of one's time at home (physical isolation), with duration of at least 6 months. This definition of social withdrawal was based on theoretically and empirically derived criteria developed for hikikomori (1, 17). Controls consisted of patients with ASD but without current social withdrawal; going out almost every day (more than 5 days a week) spending most of their time outside of their house, and having continuous direct communication with people beside their family members. Sixteen participants met the criteria for hikikomori according to the semi-structured interview.

### 2.2. Measures

Through the semi structured interview, participants were asked of the height, weight, household income, educational background, cohabitation, past history of hikikomori, and comorbidities. All comorbidities were surveyed, especially atopic dermatitis.

#### 2.2.1. Self-administered rating scales

All participants answered several self-administered rating scales; 25-Item Hikikomori Questionnaire (HQ-25) (18), Patient Heath Questionnaire 9 (PHQ-9) (19), 22-Item Tarumi's Modern-Type Depression Trait Scale (TACS-22) (9), Liebowitz Social Anxiety Scale Japanese Version (LSAS-J) (20), Mini Social Phobia Inventory (Mini SPIN) (21), Preference for Solitude Scale (PSS) (22), Internet Addiction Test (IAT) (23), and Adolescent/Adult Sensory Profile (AASP) (24). To assess symptoms of hikikomori, we used the HQ-25. In order to assess depressive symptoms, PHQ-9 and TACS-22 were used. TACS-22 measures the depression level of modern type depression (MTD). LSAS and MINI-SPIN measures the symptoms and severity of social anxiety. PSS assesses preference for solitude. IAT shows the level of internet dependence. AAAP measures sensory processing tendencies, and is categorized into four categories (low registration, sensory seeking, sensory oversensitivity, and sensory avoidance).

#### 2.2.2. Blood biomarkers

Measured blood biomarkers were as follows: serum HDL-C, LDL-C, serum total bilirubin, UA, hsCRP, and plasma FDP. These biomarkers were adopted because all items could be easily measured in normal clinical settings. Although tested samples were non-fasting venous blood, effects of meal on serum LDL-C and HDL-C were thought to be negligible.

#### 2.2.3. Statistical analyses

We used unpaired Student's *t*-test and Mann-Whitney *U*-test to compare the group difference. Next, we used stepwise binomial logistic regression to examine associations between candidate predictor variables (self-administered rating scales including subscales and blood test results) and presence of co-occurring social withdrawal. All analyses were conducted using IBM SPSS 24 Advanced Statistics for Mac OS with two-sided alpha = 0.05. The logistic regression model was used to calculate the adjusted odds ratio (OR) with 95% confidence interval (CI) for risks of hikikomori associated with ASD. The multivariate logistic regression model was performed with adjustments for all potential confounding factors as listed in Tables 1–**3**.

**Table 1:** Patient characteristics and demographics.

	**ASD with hikikomori**	**ASD without hikikomori**		
	**Mean (SD)**	**Mean (SD)**	**Student's t-test**,	
**Variables**	***N*** = **16**	***N*** = **23**	***U-test, or** χ**2***	***P*** **-value**
Age (range)	37.4 [23-57]	33 [20-48]	*t* = −1.4	0.15
**Autism spectrum quotient (AQ)**				
AQ-J	36.3 (6.0)	33.2 (6.0)	*t* = −1.59	0.12
Social skills	8.6 (1.5)	7.4 (2.5)	*U* = 234	0.16
Attention switching	7.19 (1.6)	7.1 (1.6)	*t* = −0.19	0.85
Attention to details	6.19 (2.1)	5.8 (2.7)	*t* = −0.47	0.66
Communication	7.75 (1.8)	6.7 (2.6)	*t* = −1.43	0.19
Imagination	6.6 (1.9)	6.1 (1.8)	*t* = −0.71	0.48
**Autism Diagnostic Observation Schedule (ADOS)**				
Communication	3.2 (1.2)	3.8 (1.1)	*t* = 1.24	0.22
Reciprocal social interaction	7.2 (1.1)	6.7 (1.9)	*U* = 84.5	0.67
Communication and RSI Total	10.4 (2.1)	10.5 (2.7)	*t* = 0.28	0.98
Imagination/Creativity	0.8 (0.8)	1.1 (1.4)	*t* = 0.77	0.45
Stereotyped behaviors and restricted interests	0.4 (1.0)	0.7 (0.9)	*t* = 0.51	0.62
**WAIS-III**				
VIQ	107 (16.6)	107.1 (9.7)	*U* = 180	0.66
PIQ	93.6 (19.5)	95.3 (15.8)	*t* = 0.28	0.77
FIQ	101.1 (18.9)	101.4 (12.0)	*U* = 183	0.59
**Education level**				
Bachelor's degree or higher	7 (43.5%)	14 (62%)	χ^2^ = 1.1	*P* = 0.34
Other (high school, less than high school, college)	9 (56.5%)	9 (38%)		
**Cohabitaion**				
Living alone	7 (43%)	8 (35%)	χ^2^ = 0.03	0.86
Not living alone	9 (57%)	15 (65%)		
**Household income/year**				
Below 2.5 million	8 (50%)	10 (43%)	χ^2^ = 0.16	*P* = 0.75
Above 2.5 million	8 (50%)	13 (57%)		
Atopic dermatitis	*N* = 8 (50%)	*N* = 4 (17%)	χ^2^ = 6.4	0.01
BMI, mean (SD)	24.8 (5.6)	23.5 (4.6)	*t* = −0.80	0.43
Alcohol consumption	*N* = 4 (25%)	*N* = 8 (35%)	χ^2^ = 0.42	0.52

BMI, body mass index.

## 3. Results

A total of 39 participants participated in the study. Thirty-nine met the diagnosis of ASD. Through structured interview, 16 ASD patients were in the hikikomori state (“cases”), and 23 ASD patients were not in a hikikomori state (“controls”). Seventeen patients were taking medications on a regular basis, three using medications on a needed basis, and 22 patients had no medications in terms of psychotropic drugs. There were no significant differences by regular basis medication in both groups (*p* = 0.0.21). Most medications were consisted of sleep medication. Secondly, anxiolytic drugs, and small dose of antipsychotic drugs were prescribed for minor social anxiety below diagnostic criteria and irritability. Three patients had regular prescription of antidepressants—mainly prescribed for secondary depression developed by ASD symptoms. Subject backgrounds in the hikikomori and control (non-hikikomori) groups were similar for age, and severity of ASD characteristics (AQ-J, ADOS), and FIQ.

Demographic and characteristic data of are shown in Table 1. As of educational background (*p* = 0.34) and income of the household (*p* = 0.86), there were no significant difference in both groups. Body mass index (BMI) did not differ between the two groups (*p* = 0.43). Fifty percent of the hikikomori cases had atopic dermatitis, showing significant difference to control cases (*p* = 0.01). There were no significant differences in alcohol consumption habits (*p* = 0.52).

The results of the self-administered questionnaires are shown in Table 2. As for the HQ-25, cases showed higher total scores (*p* = 0.002). In addition, the cases differed on subscale of higher socialization (*p* = 0.01) and higher isolation (*p* < 0.001). PHQ-9 scores were significantly higher in cases (*p* < 0.001), showing higher signs of depression. TACS-22 showed cases had higher overall scores (*p* < 0.001) and all of the sub-scales: avoidance (*p* = 0.003), self-esteem (*p* = 0.002), and complaint (*p* < 0.001). LSAS scores were higher in the performance category of fear (*p* = 0.05) and avoidance (*p* = 0.04), and total scores (*p* = 0.04). MINI-SPIN showed no significant difference between the two groups (*p* = 0.15). PSS showed higher values in cases (*p* = 0.02), but no difference was found in the IAT (*p* = 0.65). AASP scores in low registration (*p* = 0.04), sensory sensitivity (*p* = 0.002), and sensory avoidance (*p* = 0.003) showed significantly higher scores in hikikomori cases.

**Table 2:** Self-rated questionnaires.

	**ASD with hikikomori**	**ASD without hikikomori**	
	**Mean (SD)**	**Mean (SD)**		
	***N*** = **16**	***N*** = **23**	***Student's t-test, or U-test***	***P*** **-value**
**HQ-25**				
HQ-25 total	69.6 (14.2)	53.2 (16.1)	−3.35	0.002
HQ-25 socialization	33.1 (7.0)	26.6 (7.5)	−2.76	0.01
HQ-25 isolation	25 (6.6)	15.5 (4.1)	*U* = 325	<0.001
HQ-25 ES	11.5 (6.7)	11.1 (5.7)	−0.20	0.84
HQ-25 motivation	8 (3.5)	7.5 (0.8)	−0.44	0.67
PHQ-9 total	14.9 (6.4)	6.8 (4.9)	−4.26	<0.001
**TACS 22**				
TACS total	63.1 (12.0)	47.3 (10.7)	−4.24	<0.001
TACS avoidance of social roles	30.3 (5.6)	24.3 (6.0)	−3.19	0.003
TACS self esteem	17.3 (3.8)	13.1 (3.8)	−3.37	0.002
TACS complaint	15.6 (5.6)	9.9 (4.1)	−3.45	<0.001
LSAS total	73.4 (33.2)	52.3 (27.8)	−2.09	0.04
LSAS avoidance	33.5 (17.2)	22.7 (14.0)	−2.07	0.04
LSAS fear	39.8 (16.7)	29.5 (15.3)	−1.97	0.05
Mini-SPIN total	6.9 (4.3)	5 (3.8)	−1.45	0.15
Preference for solitude total	10.3 (2.5)	7.8 (3.4)	−2.60	0.02
Internet Addiction Test (IAT)	47.1 (23.2)	43.8 (20.2)	−0.45	0.65
**Adolescent/Adult Sensory Profile (AAAP)**				
Low registration	42.25 (10.3)	35.5 (9.4)	−2.10	0.04
Sensory exploration	30.2 (4.6)	33.5 (6.7)	1.85	0.09
Sensory sensitivity	51.1 (13.7)	36.7 (8.8)	*U* = 288.5	0.002
Sensory avoidance	50.3 (12.6)	39.0 (9.0)	−3.05	0.003

HQ-25, 25-Item Hikikomori Questionnaire; TACS22, 22-Item Tarumi's Modern-Type Depression Trait Scale: Avoidance of Social Roles, Complaint, and Low Self-Esteem; PHQ-9, Patient Health Questionnaire 9; Mini-SPIN, Mini Social Phobia Inventory; LSAS, Liebowitz Social Anxiety Scale.

**Figure 1:**
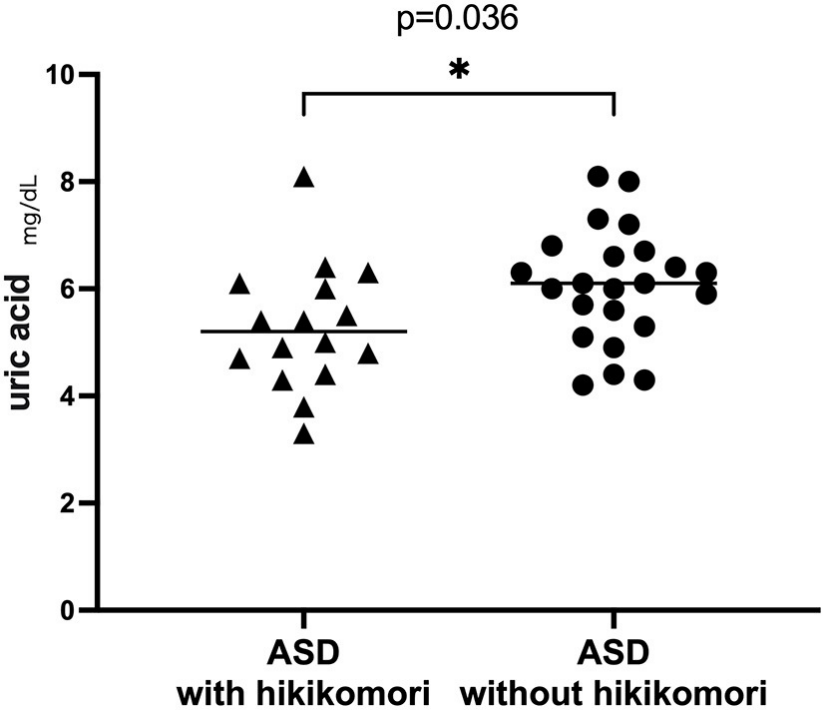
Uric acid in hikikomori and non-hikikomori cases.

The results of blood tests are shown in Table 3. In terms of blood tests, cases significantly differed in uric acid level (UA). Serum UA were lower in cases (5.27 mg/dl) than controls (6.05 mg/dl) (*p* = 0.036) (Figure 1). Other biochemical data from the blood test were similar in both groups.

**Table 3:** Blood biomarkers.

	**ASD with hikikomori**	**ASD without hikikomori**		
	**Mean (SD)**	**Mean (SD)**		
	***N*** = **16**	***N*** = **23**	***Student's t-test, or U-test***	***P*** **-value**
HDL-C (mg/dl)	51.4 (11.5)	58.4 (14.7)	1.67	0.12
LDL-C (mg/dl)	115.5 (26.9)	104.7 (25.2)	−1.26	0.21
T-Chol (mg/dl)	166.8 (27.1)	163.1 (26.6)	−0.43	0.67
FIB (mg/dl)	259.0 (63.5)	242.8 (53.7)	−0.84	0.39
FDP (mg/dl)	0.88 (1.6)	0.39 (1.1)	*U* = 207.5	0.51
T-Bil (mg/dl)	0.47 (0.2)	0.54 (0.2)	1.03	0.30
D-Bil (mg/dl)	0.16 (0.1)	0.19 (0.1)	*U* = 147.5	0.30
UA (mg/dl)	5.28 (1.2)	6.01 (1.1)	2.18	0.036
hsCR (mg/dl)	646.6 (630.7)	540.0 (540.0)	−0.68	0.48

HDL-C, high density lipoprotein cholesterol; LDL-C, low density lipoprotein cholesterol; T-Chol, total cholesterol; FIB, fibrinogen; FDP, fibronogen degradation products; T-Bil, total bilirubin; D-Bil, direct bilirubin; UA, uric acid; hsCRP, high sensitivity C-reactive protein.

Logistic regression analysis revealed that HQ-25 subscale “isolation” (OR = 2.46, 95% CI: 1.17–5.19, *p* = 0.018) and UA (OR = 0.054, 95% CI: 0.016–0.054, *p* = 0.016) were predictors of hikikomori.

## 4. Discussion

From this study, hikikomori cases in ASD patients significantly had atopic dermatitis as a common comorbidity. Significantly lower uric acid level, higher depressive symptoms, higher preference of solitude, higher social anxiety scores, and higher sensory sensitivity, avoidance, and lower registration were also seen in hikikomori cases with ASD.

Interestingly, in the present study, ASD accompanied by atopic dermatitis was significantly more common in the hikikomori group. There are many earlier studies reporting of atopic dermatitis and ASD as a comorbidity, and yet the common pathogenesis between the two disorders is still uncertain (12). Prior studies have shown that genetic variants in Stat6, which is crucial for the regulation of Th2 immune responses, are associated with atopy. Furthermore, Stat6 is highly expressed in the central nervous system and has been found to play an important role in the etiology of several neuropsychiatric disorders, including ASD (25). Previously, Zimmerman et al. has reported the coexistence of high levels of inflammatory cytokines in both atopy and neuropsychiatric disorders, while other reports have shown atopic symptoms itself being associated with depression and anxiety disorders (26). In addition, atopic symptoms have been reported to be associated with sensory sensitivity and avoidance from childhood (27). From the results of our study, the combination of ASD and atopy may have a significant impact on the state of hikikomori. Although further research is necessary, there may be a complex interaction of atopic dermatitis, ASD, and hikikomori.

HQ-25 was significantly higher in the hikikomori group than in the controls. This ensures the validity of the semi-structured interviews we conducted. In the present study, self-administered rating scales indicated that ASD in the hikikomori group showed a stronger degree of depression (PHQ-9 and TACS22) and a higher degree of social anxiety (LSAS) compared to ASD in the non-hikikomori group. In addition, the study showed that sensory abnormalities, which is a characteristic often associated with ASD, were highly prevalent in the hikikomori condition.

Depression and social anxiety is widely known to comorbid with ASD (28). Although none of the subjects in this study met the clinical diagnosis of depression or social anxiety disorder (DSM-5), the hikikomori group had higher tendencies of depression and social anxiety on self-administered scales. This suggests that in ASD, even if below the diagnostic threshold, being in a mental state likewise of depression such as low motivation, and/or having a high sense of nervousness when communicating with others due to social anxiety, can easily lead to a state of hikikomori.

Modern-Type Depression (MTD) is a concept that has been attracting attention in Japan since around 2000, when interest in hikikomori increased (29). It has been pointed out that MTD is characterized by a situation-dependent depressed mood, extrapunitive, strong avoidance tendencies (9), and leads easily to social withdrawal (30, 31). Katsuki et al. reported that among 103 persons who were hikikomori, the total score o fTACS-22, a self-administered questionnaire which measures the traits of MTD, was higher in the group which exceeded the cutoff value of the AQ (7). In the present study, TACS-22 scores and all of the sub-scales (avoidance of social roles, self-esteem, and complaint) were significantly higher in the hikikomori group of ASD. This result suggests that among ASDs, a group with MTD-like characteristics may be more likely to be associated with hikikomori.

In the present study, AQ and ADOS which mainly measures ASD characteristics were set to have no significant difference between the hikikomori and non-hikikomori groups. In AASP, which measures sensory abnormality—a typical ASD characteristic, sub-scales measuring low registration, sensory sensitivity, and sensory avoidance, were significantly higher in the hikikomori group. This indicates that among ASD characteristics, certain sensory abnormalities may represent a characteristic that is likely to be associated with hikikomori.

Another interesting finding was the low level of uric acid (UA) in the hikikomori group, which showed a possibility of becoming a biomarker in the hikikomori state. In another previous study of hikikomori, low levels of UA in males and low levels of HDL-C in females were shown among hikikomori compared to healthy volunteers (32). In this present study, male ASD patients with hikikomori also showed significantly lower blood uric acid levels. Furthermore, the results of a binomial logistic regression analysis with presence or absence of hikikomori as the dependent variable indicated that low uric acid levels could be a risk for hikikomori. Uric acid is an endogenous antioxidant which in known to decrease oxidative stress (33), while oxidative stress is known to decrease serotonin levels in the brain (34). An association between avoidant personality disorders and serotonin nervous system regulations has also been reported in earlier studies (35). Therefore, in light of the present results, a pathway which starts from low uric acid, leading to increased oxidative stress which disturbs the serotonergic system, may possibly be the underlying biological basis of ASD with hikikomori condition.

The purpose of this study was to clarify the factors that tend to lead ASD to hikikomori, from psychological and biological aspects. It is hoped that these findings lead to preventions of ASD from leading to hikikomori, and the development of possible new perspectives for physicians and other professionals, for future treatment of both ASD and hikikomori.

### 4.1. Limitations

There are several limitations to this present study. First, this study is a relatively small study. Therefore, further research with more cases and controls is desirable. However, this study is the first survey of hikikomori among individuals with ASD, most of which were strictly diagnosed as ASD by ADOS. Secondly, for many patients with hikikomori, coming to the hospital for outpatient visiting itself may be a strain, and there is a possibility of patients with a more severe level of hikikomori still not being helped from social or medical help. In this study, we have evaluated patients which have already been connected to medical help, which may not evaluate the true severity of ASD and hikikomori. Third, although there were several interesting biological findings, cytokines, which is likely to be related, were not measured in this study. In future studies, measurement is desired. Fourth, ASD patients often have difficulty in accurately being objective about themselves, which may possibly make self-administered questionnaires difficult. Lastly, in this study, some of the patients were taking medication. Therefore, the effect of medications could not be ruled out. We plan to increase the sample size in the future to investigate the effect of medication. Furthermore, prospective studies and studies aiming for intervention techniques is needed in the future.

### 4.2. Conclusion

The present study has suggested ASD patients with hikikomori were more likely to have higher sensory abnormalities, comorbid atopic dermatitis, lower uric acid, stronger depressive, and anxiety tendency. Evaluating and approaching these aspects are important for appropriate interventions in ASD with hikikomori. Further investigations should be conducted to validate our pilot findings.

## Data availability statement

The original contributions presented in the study are included in the article/supplementary material, further inquiries can be directed to the corresponding author/s.

## Ethics statement

The studies involving human participants were reviewed and approved by Faculty of Medicine of Showa University. The patients/participants provided their written informed consent to participate in this study.

## Author contributions

HO and TK initially designed the study. MY and HO participated in study design and statistical analyses, literature searches, and drafted the initial manuscript. RK, HY, MI, YKo, YKa, NK, and AI oversaw the data analysis and participated in data interpretation and the writing of the manuscript. All authors contributed to the article and approved the submitted version.
